# *Colocasia kachinensis*, a new species of Araceae from Myanmar

**DOI:** 10.3897/phytokeys.138.36769

**Published:** 2020-01-10

**Authors:** Shi-Shun Zhou, Rui-Chang Quan, Ren Li, Qiang Liu, Jian-Tao Yin

**Affiliations:** 1 Southeast Asia Biodiversity Research Institute, Chinese Academy of Sciences, Yezin, Nay Pyi Taw 05282, Myanmar Xishungbanna Tropical Botanical Garden, Chinese Academy of Sciences Mengla China; 2 Center for Integrative Conservation, Xishungbanna Tropical Botanical Garden, Chinese Academy of Sciences, Menglun, Mengla, Xishuangbanna, Yunnan 666303, China Southeast Asia Biodiversity Research Institute, Chinese Academy of Sciences Yezin Myanmar; 3 Yunnan Forestry Technological College, Jindian Road, Panlong District, Kunming, Yunnan 650000, China Yunnan Forestry Technological College Kunming China

**Keywords:** Araceae, *Colocasia kachinensis*, Mynamar, Holotype, *Colocasia menglaensis*

## Abstract

*Colocasia kachinensis* S.S. Zhou & J.T. Yin, is described and illustrated as a new species of Araceae from Kachin, Mynamar. The morphological characters are compared to those of other *Colocasia* species. *Colocasia kachinensis* is closely related to *C. menglaensis* J.T Yin, H. Li & Z.F. Xu, 2004, but differs from in having an erect stem, no stolons, smaller size, a different pattern of surface bristle distribution and male flowers 1–4-androus with stamens connate in truncate synandrium.

## Introduction

*Colocasia* is a genus of about 20 species distributed in tropical and subtropical Asia (Li and Boyce 2010). Currently, two sections are recognised within the genus: sect. Colocasia and sect. Caulescentes (Mayo et al. 1997). This new species belongs to section Caulescentes Engl., characterised by an erect stem.

Including the species described here, four *Colocasia* species are known in Myanmar (Kress et al. 2003): *C. affinis* Schott, *C. esculenta* (L.) Schott, *C. kachinensis* and *C. menglaensis* J.T. Yin, H. Li & Z.F. Xu. *Colocasia menglaensis* was first found in the same habitat as *C. kachinensis*.

During an expedition to Kachin in April 2016, two populations of an unusual *Colocasia* were encountered growing along the roadside in the understorey of a mountain rain forest. For the next two years, the authors monitored the in-situ population, as well as plants established in ex-situ collection and meticulously examined and documented flowering episodes of the species. The unusual *Colocasia* sp. was compared with closely allied species and the gathered evidence revealed that the species was new to science.

## Taxonomy

### Colocasia kachinensis

Taxon classificationPlantaeAlismatalesAraceae

S.S.Zhou & J.T.Yin, sp. nov.

7F197B48-D09B-53A8-A016-6895F173083B

urn:lsid:ipni.org:names:77204196-1

**Figure 1.**
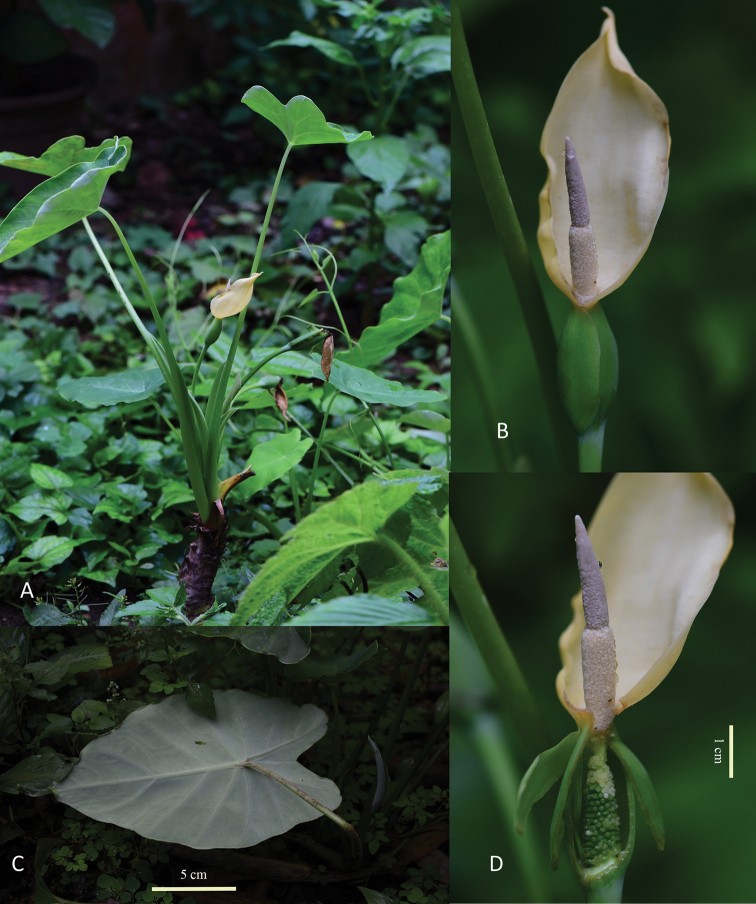
*C. kachinensis*. **A** plant **B** inflorescence **C** lower surface of leaf **D** spadix.

**Figure 2.**
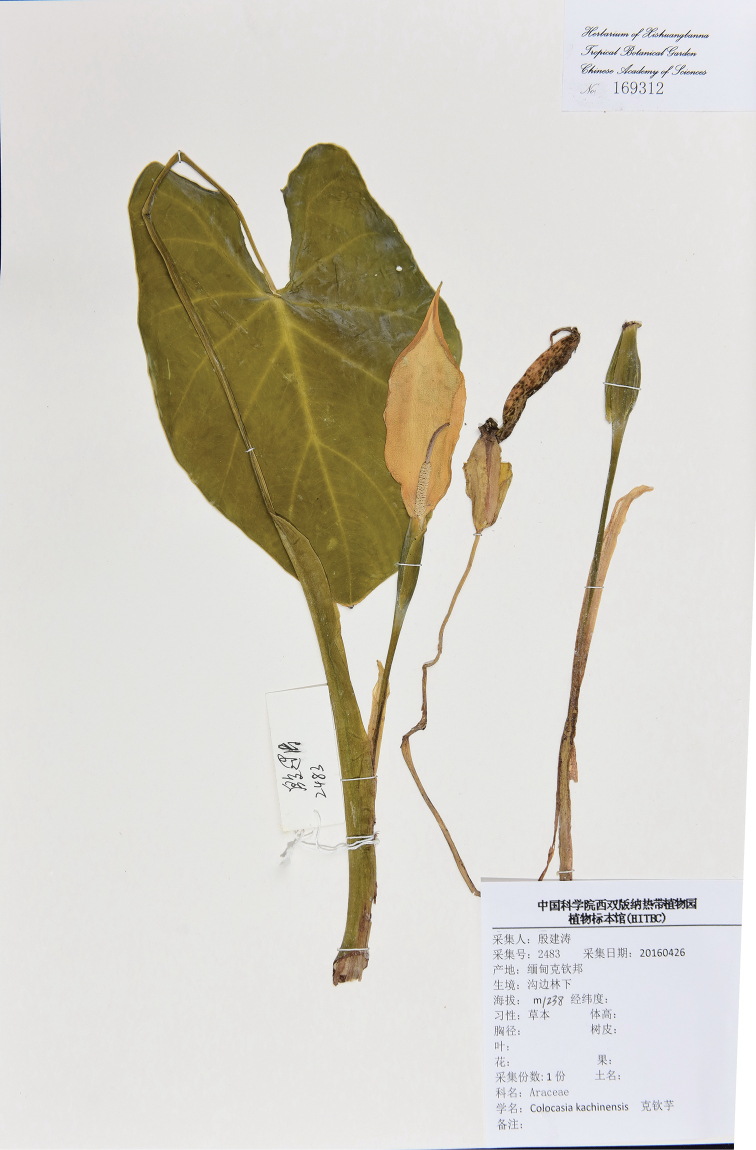
Holotype of *C. kachinensis.* See text for collection details.

**Figure 3.**
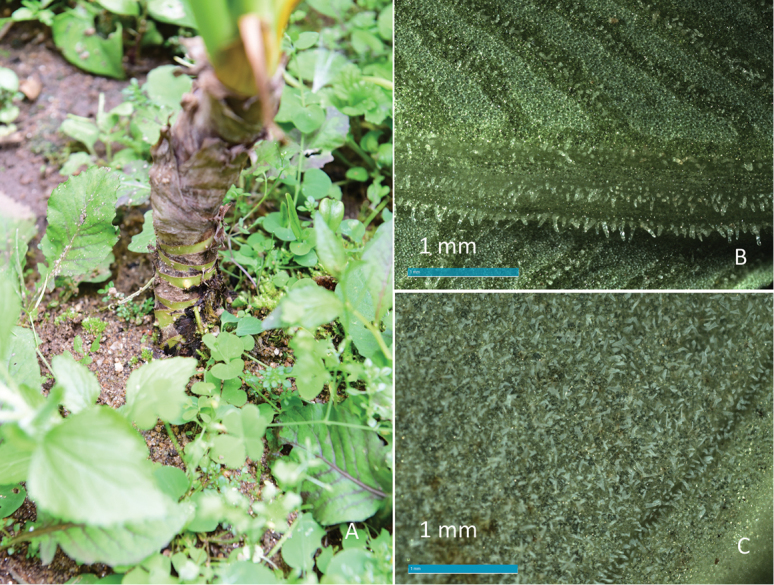
Stem of *C. kachinensis* and morphological comparison between *C. menglaensis* and *C. kachinensis*. **A** stem of *C. kachinensis***B** lower surface of leaf ×100 of *C. menglaensis***C** lower surface of leaf ×100 of *C. kachinensis*.

**Figure 4.**
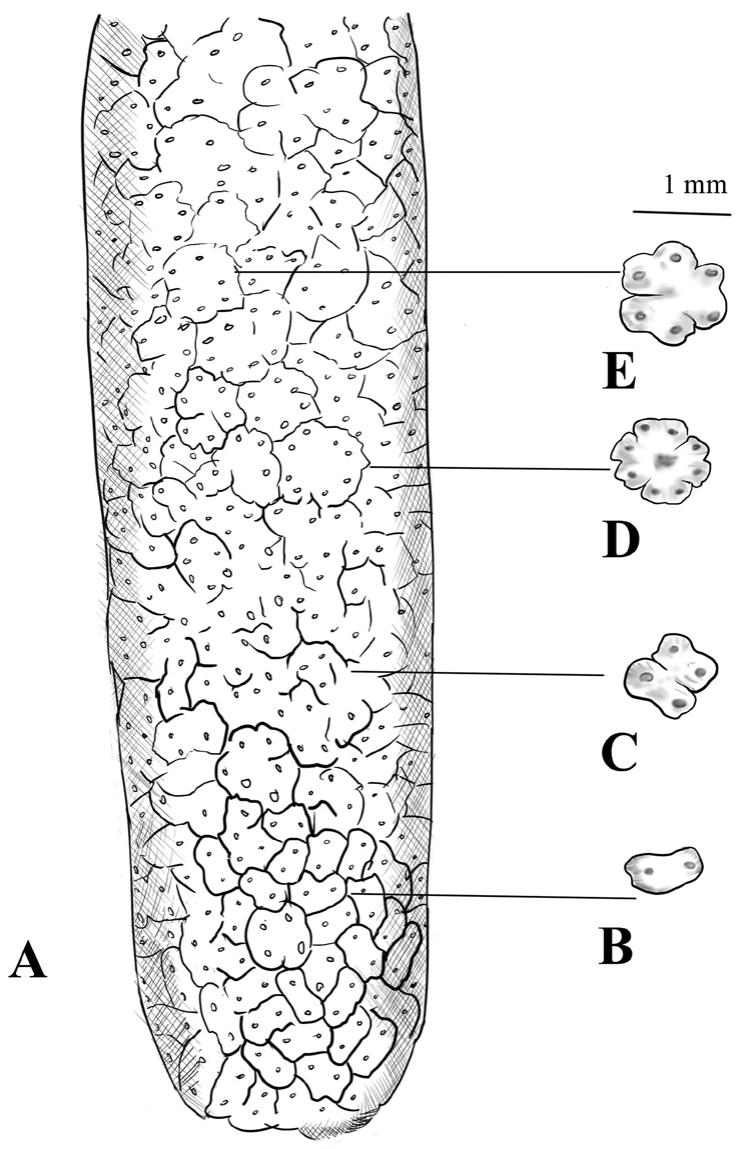
Male flower of *C. kachinensis.* Drawn by Mr. Bo Pan from the holotype. **A** Male part of spadix **B** 1-androus flower **C** 2-androus flower **D** 4-androus flower **E** 3-androus flower.

Figures 1 , 2 , 3 , 4

Colocasia sect. Caulescentes Engl.

#### Diagnosis.

The morphological characteristics of *C. kachinensis* are closely related to those of *C. menglaensis* but *C. kachinensis* differs in having an erect stem (see Fig. 3), no stolons, smaller leaf and inflorescence and glossy petiole and peduncle.

#### Type.

MYANMAR. Kachin State. Putao Township, Hponkanrazi Wildlife Sanctuary, Namse Village, 97°18'30.3"E, 27°17'49.7"N, alt. 1238 m, 26 April 2016, Jian-Tao Yin 2483 (Fig. 2) (holotype, HITBC!, isotype: HITBC!)

#### Description.

Terrestrial perennial herbs with an erect stem. Plant 54 cm high; erect stem 12 cm long, 3 cm in diam. Leaves 3–4; petiole cylindric, pale greenish, glossy, 32 cm long, 0.6 cm in diam., sheath 16 cm long, 6 cm in diam.; leaf blade oblong-ovate, peltate, 18 cm long, 12 cm wide, upper surface glossy green, lower surface greyish-white; primary lateral veins pinnate, 5 pairs, pale green on upper surface, white and raised on the lower surface. Inflorescences (1-)3(-4) emerging when the leaves unfold, 27 cm long; peduncle cylindrical, pale green, glossy, 17 cm long, 0.5 cm in diam. Spathe constricted in the lower third, lower convolute part (tube) pale green, farinose, 3.5 cm long, 1.5 cm in diam., nearly cylindrical; lamina oblong-lanceolate, erect during early blooming period, pale yellow, 6.5 cm long, 3 cm wide. Spadix 7 cm long, female zone 2.5 cm long, 0.8 cm in diam.; sterile zone between female and male zones, cylindrical, white, 0.8 cm long, 0.3 cm in diam.; male zone, white, 2 cm long, 0.6 cm in diam.; appendix white, long conical, wrinkled, 2 cm long, 0.4 cm in diam. Flowers unisexual, perigone absent. Male flower: 1–4-androus, stamens connate in truncate synandrium, thecae lateral, oblong-lineal, dehiscing by apical pore. Female flower: ovary ovoid to oblong, 1 mm long, unilocular; ovules many, 42–58, n = 2, fusiform, translucent; placentae 3–5, parietal; stylar region absent; stigma discoid-capitate; berry not seen.

#### Phenology.

Flowering in March to April. Fruiting unknown.

#### Distribution and habitat.

*C. kachinensis* is so far known from a single population in Kachin State, northern Myanmar, where it grows in humid dense mountain rain forest (cover degree 70%) at alt. 1100–1400 m. In the same habitat, other plants encountered were *C. menglaensis*, *Liquidambar excelsa*, *Terminalia myriocarpa*, *Caryota urens*, *Magnolia* sp., *Musa itinerans*, *Saprosma ternate*, *Dendrocalamus* sp., *Phrynium rheedei*.

#### Etymology.

The species is named after the holotype region, Kachin State, Myanmar.

#### Additional examined specimens (Paratype).

MYANMAR. Putao Township, Kachin State, alt. 1300 m, 26 April 2016, Jian-Tao Yin 2482 (paratype: HITBC!)

## Discussion

*Colocasia kachinensis* is similar to *C. menglaensis*, described by Yin et al (2004), because they both have similar pubescent leaves. It differs from the latter by having (i) an erect stem (see Fig. 3), (ii) no stolons, (iii) smaller leaf and inflorescence and (iv) glossy petiole and peduncle.

*Colocasia menglaensis* and *C. kachinenis*, which were introduced from Myanmar, have been grown in a small yard of Xishuangbanna Tropical Botanical Garden, Yunnan China. We then collected leaves of two species for observation. The lower surface of the leaf blade, observed with a 100× magnification, also shows that *C. kachinensis* differs from *C. menglaensis*. The short bristles of the former are uniformly distributed on the abaxial surface of the leaf, while those of *C. menglaensis* are concentrated on the veins on the abaxial surface (see Fig. 3).

*Colocasia kachinensis* is also different from other species in this genus by having 1–4-androus male flowers, with stamens connate in truncate synandrium (see Figure 4). In the genus of *Colocasia*, one usually finds 3–6-androus male flowers, with stamens connate in ± truncate synandrium (Mayo et al. 1997). Further differences are listed in Table 1.

**Table 1.** Morphological differences between *C. kachinensis* and *C. menglaensis*.

Characters	*C. kachinensis*	*C. menglaensis*
Rhizome	erect	decumbent
Stolon	none	6–10 per plant,15–20 cm long, 4 mm in diam.
Petiole	glossy	pubescent
Blade	18 × 12 cm	40 × 25 cm
Primary lateral vein	5 pairs	7–9 pairs
Penducle	glossy	pubescent
Spathe lamina	milk yellow, 6.5 × 3 cm	yellowish, 13–18 × 4–6 cm
Female zone	2.5 cm long, 0.8 cm in diam.	2 cm long, 1 cm in diam.
Male zone	2 cm long, 0.6 cm in diam.	3.5 cm long, 0.7 cm in diam.
Appendix	2 cm long, 0.4 cm in diam.	3.5 cm long, 0.5 cm in diam.
Male flower	1–4-androus	8–11-androus

## Supplementary Material

XML Treatment for Colocasia kachinensis
